# Quantifying the Interaction between Copper-Zinc Superoxide Dismutase (Sod1) and its Copper Chaperone (Ccs1)

**DOI:** 10.4172/jpb.1000473

**Published:** 2018-05-14

**Authors:** Stefanie D Boyd, Li Liu, Lee Bulla, Duane D Winkler

**Affiliations:** Department of Biological Sciences, The University of Texas at Dallas, 800 W. Campbell Road, Richardson, TX 75080, USA

**Keywords:** Metallo-chaperones, Copper, Sod1, Metallo-enzymes

## Abstract

Immature copper-zinc superoxide dismutase (Sod1) is activated by its copper chaperone (Ccs1). Ccs1 delivers a single copper ion and catalyzes oxidation of an intra-subunit disulfide bond within each Sod1 monomer through a mechanistically ambiguous process. Here, we use residue specific fluorescent labeling of immature Sod1 to quantitate the thermodynamics of the Sod1•Ccs1 interaction while determining a more complete view of Ccs1 function. Ccs1 preferentially binds a completely immature form of Sod1 that is metal deficient and disulfide reduced (E, E-Sod1^SH^). However, binding induces structural changes that promote high-affinity zinc binding by the Ccs1-bound Sod1 molecule. This adds further support to the notion that Ccs1 likely plays dual chaperoning roles during the Sod1 maturation process. Further analysis reveals that in addition to the copper-dependent roles during Sod1 activation, the N- and C-terminal domains of Ccs1 also have synergistic roles in securing both Sod1 recognition and its own active conformation. These results provide new and measurable analyses of the molecular determinants guiding Ccs1-mediated Sod1 activation.

## Introduction

Copper-zinc superoxide dismutase (Sod1) is an abundant antioxidant enzyme that readily converts superoxide anions into peroxide and molecular oxygen [2O_2_^−^+2H^+^ → H_2_O_2_+O_2_] [1]. Sod1 is subject to numerous post-translational modifications that convert the nascent polypeptide into its extremely stable homodimeric native conformation [2,3]. Immature Sod1 is targeted by the copper chaperone for Sod1 (Ccs1), which delivers the copper ion to the active site and catalyzes formation of an intra-subunit disulfide bond [4–6]. Zinc is assumed to be diffusively acquired by the environment and bound at the “zinc-site” adjacent to the active site [6–8]. More recent evidence suggests that Ccs1 may have additional molecular chaperone-like roles during Sod1 activation [9–11]. Ccs1 has evolved an activity specific only for Sod1 and Sod1 cannot be activated by other copper chaperones [4,12].

The Ccs1 molecule contains three functional domains (D1, D2 and D3) [12]. Starting at the N-terminus, D1 is structurally homologous to the Atx1 copper chaperone from yeast and contains the same MxCxxC copper-binding motif [13–15]. Domain 1 is required for action under strict copper limiting conditions which suggests a role in copper acquisition from the influx copper transporter Ctr1 or other cytosolic copper scavenging molecules like reduced glutathione (GSH) [12,16]. The second domain (D2) has sequence and fold similar to that of Sod1 [13]. Overwhelming evidence advocates a direct role in Sod1 recognition and binding during the activation event by taking advantage of the same interaction interface exploited by the Sod1 homodimer [9,17]. Numerous high-resolution structures of Ccs1 and Sod1•Ccs1 complexes have been determined and show D2-mediated homo-/heterodimers [13,18,19]. Domain 3 is the most conserved portion of the Ccs1 molecule and contains an invariant CxC copper-binding motif that is critical for complete Sod1 activation [4,12]. This short domain is likely unstructured in the absence of copper binding and/or Sod1 interaction [13]. Together, the structural architecture of Ccs1 creates a highly specific and efficient chaperoning molecule ensuring efficient Sod1 activation while protecting the cell from aberrant copper-related reactions.

**Figure 1:**
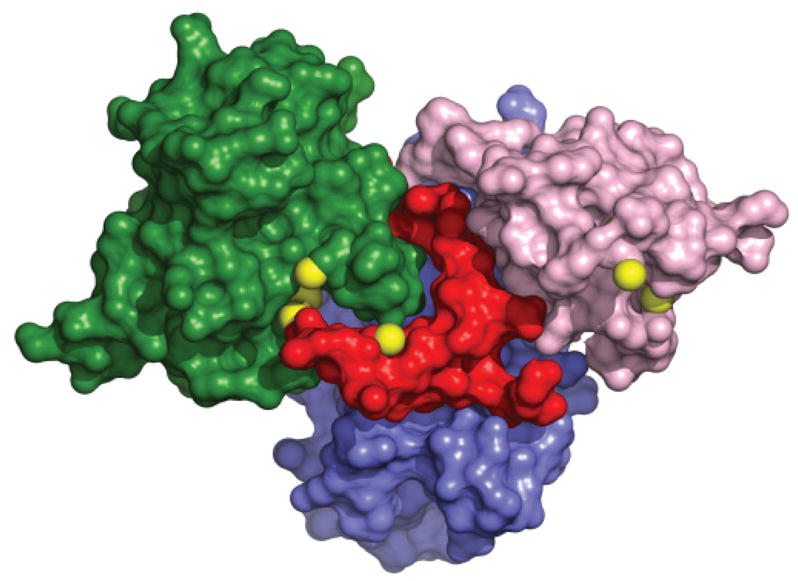
Sod1•Ccs1 heterodimeric complex structure. The Ccs1 surface is colored by domain: D1 (pink) and D2 (purple) and D3 (red) are shown with the cysteines of the MxCxxC and CxC motifs indicated by yellow spheres. The Sod1 surface is colored green and the critical cysteines that form an intramolecular disulfide bond as yellow spheres. The Sod1 β-barrel engages Ccs1 D2 and this interface is expanded by a notch-into-groove type interaction between the disulfide loop of Sod1 and Ccs1 D3.

The mechanistic details of the Ccs1-mediated Sod1 activation process remain ambiguous. Discrepancies in the literature coming from both structural and functional studies contribute greatly towards this uncertainty [9, 12,18,20–24]. We recently reported the crystal structure of a Sod1•Ccs1 heterodimeric complex highlighting an extended interaction interface between the two molecules (Figure 1) [18]. The structure is suggestive of undescribed roles for Ccs1 D1 and D3 during Sod1 recognition, binding and activation by Ccs1. In conjunction, reports from numerous independent groups, including our own, have shown evidence for additional chaperoning roles for Ccs1 involving disease-causing mutant forms of Sod1 [10,11,18].

A clearer understanding of the Sod1•Ccs1 interaction and mechanism of Ccs1 action is important not only for the fields of copper trafficking and metallo-enzyme function, but also for the realm of Sod1-linked amyotrophic lateral sclerosis (ALS). An increasing amount of data points to metal deficient disulfide reduced (E, E-Sod1^SH^) forms of Sod1 mutants making up the majority of protein within the aggregates found in transgenic mice models and ALS patient autopsies [25–29]. These results strongly suggest that the Sod1/Ccs1 transaction cannot be completed in these instances, though the point terminating the process may be different among the great variety of disease causing Sod1 mutants [7,9,30].

## Material and Methods

### Sod1 and Ccs1 cloning, expression and purification

DNA fragments encoding yCcs1 were generated by polymerase chain reaction (PCR) from plasmids supplied by the Hart lab at UTHSCSA. Ccs1 constructs were cloned into a pkA8H or pHAT4 vector, which both contain an inducible *LacZ* promoter, an 8x-N-terminal His-tag, and a tobacco etch virus (TEV) cleavage site. Site specific amino acid changes in either Sod1 or Ccs1 were done via quick-change mutagenesis. Sod1 and Ccs1 proteins were expressed in *Escherichia coli* BL21 (DE3) pLysS. Cells containing these expression plasmids were grown in LB media at 37°C to an OD_600_ of 0.6 to 0.8. After induction with IPTG, the cells were transferred to 37°C for an additional 4 hours before being harvested. Overexpressed proteins were purified using a HisTrap HP Ni^2+^ affinity column purchased from GE. After purification, the 8x-His-tag was removed from the proteins using TEV protease produced in-house and engineered to contain its own non-cleavable 6x-His tag. After digestion, the cleaved His-tag and TEV protease are removed from the sample by a final pass through the nickel column. This procedure leaves a two residue (Gly-His) extension on the N-terminus of the purified protein. The metal content of purified Ccs1 proteins was determined using inductively-coupled plasma mass spectrometry (ICP-MS) here at UTD.

### Labeling of Sod1 with fluorescent labels

The Alexa-546 C5-malemide dyes (Invitrogen) were mixed at a 1:1 ration with purified zinc-bound C146S Sod1 in a buffer containing 20 mM Tris pH 7.5, 300 mM NaCl and 0.5 mM TCEP. The mixture is allowed to incubate in the dark at 4°C overnight. The amount of protein is not critical, but the concentration of the protein is kept near 1–5 μM to avoid aggregation events. The next morning the protein is concentrated in spin concentrators to both lower the total volume and remove some of the excess dye. The sample is then loaded onto a Superdex 200 SEC column from GE. The column is wrapped in foil to keep the sample in the dark during the separation. The sample is fractionated and then run on SDS-PAGE. The excess dye elutes near the bed volume of the column and the protein sample is run on UV-vis at 280 nm to find the final concentration.

### Microplate-based binding assays

The microplates are prepared, and titration experiments are setup as previously described with a few alterations [31]. The binding experiments are performed in a reaction buffer containing 20 mM Tris pH 7.5, 150 mM NaCl, 1 mM TCEP and 0.01% octyl glucoside and CHAPS. The plates are imaged using a GE Typhoon FLA 9500 using filters specific for 488 nm excitation. The fluorescence change is then quantified using ImageQuant TL and then analyzed using GraphPad Prism.

### Spectroscopic Zinc release assays

The setup, execution and analysis of these experiments were completed nearly identical to our previous work [9]. One alteration for the some of the runs is the addition of Ccs1 to the samples along with Sod1. The Ccs1 is added in a 1:1 ratio to the Sod1. The mixtures are allowed to incubate for 20–30 minutes before starting measurements. We are using a NanoDrop 2000 spectrophotometer from ThermoFisher for the measurements at 500 nM.

### Sod1 activity assays

Sod1 was stripped of metals by dialysis in 10 mM EDTA pH 3.8 followed by 1 mM EDTA pH 5.5. Dialysis in 10 mM EDTA buffer was done for 8 hours followed by dialysis in the 1 mM EDTA buffer overnight (all at 4°C). Trace metals from tubes, tips, and glassware for activation experiments were removed by treating with 0.5 mM EDTA. Copper loading was performed under anaerobic conditions. Ccs1 protein was incubated with 1 molar equivalent of Cu1-(CH_3_CN_4_) PF6 (Strem Chemicals) for 2 hours in 50 mM Tris, pH 8, 10 mM TCEP, and 150 mM NaCl. After incubating the protein with copper overnight at 4°C in the anaerobic box, unbound copper was removed by washing the protein with degassed buffer. Activity assay reactions were set up in an aerobic environment with a 1:5 molar ratio of Sod1 to Ccs1. Sod1 was added to reaction buffer (50 mM Tris pH8, 100 mM NaCl, 0.5 mM TCEP, 20 uM Zn_2_SO_4_, and 200 uM BCS) before Cu1-Ccs1 addition. Samples were incubated for 20 minutes prior to visualization using the Nitro Blue Tetrazolium (NBT) gel method.

## Results

### Fluorescent-labeling of immature Sod1

**Figure 2:**
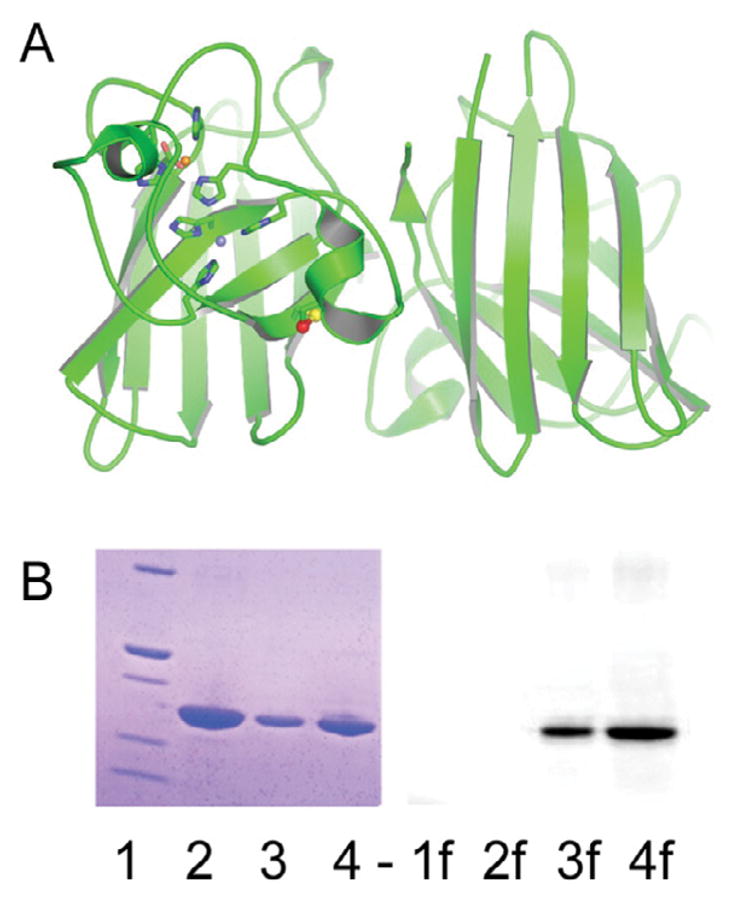
Sod1 fluorescent labelin: The fluorescent label attachment site a requirement for measuring the unusually tight binding affinities (C57) is shown as a red sphere on the Sod1 monomer (A). Panel B shows a coomassie stained image (1–4) and fluorescently imaged (1f-4f) SDS-PAGE gel of Alexa 546-labeled Sod1. Fluorescence emission from labeled Sod1 is seen in 3f and 4f.

The yeast form of Sod1 has four cysteine residues, two of which are involved in the essential intra-subunit disulfide bond (C57–C146). The two additional cysteines (C6 and C111) are both found within the Sod1•Sod1 homodimeric interface. We use a C146S mutant to specifically label Sod1 with an Alexa-546 fluorescent dye via a maleimide linkage (Figure 2A). Using a zinc-bound dimeric form of Sod1 (E, Zn-Sod1^SH^) ensures that C6 and C111 are inaccessible during the fluorescent labeling process. The labeled protein behaves essentially identical to the unlabeled disulfide-reduced Sod1 and shows no signs of aggregation or other non-native behavior in solution (Figure 2B). The advantage of this fluoro-labeled-Sod1 protein is the ability to monitor the protein at extremely low concentrations, a necessity for measuring its high-affinity interactions with Ccs1 [32].

### Ccs1 preferentially binds a completely immature form of Sod1

**Figure 3:**
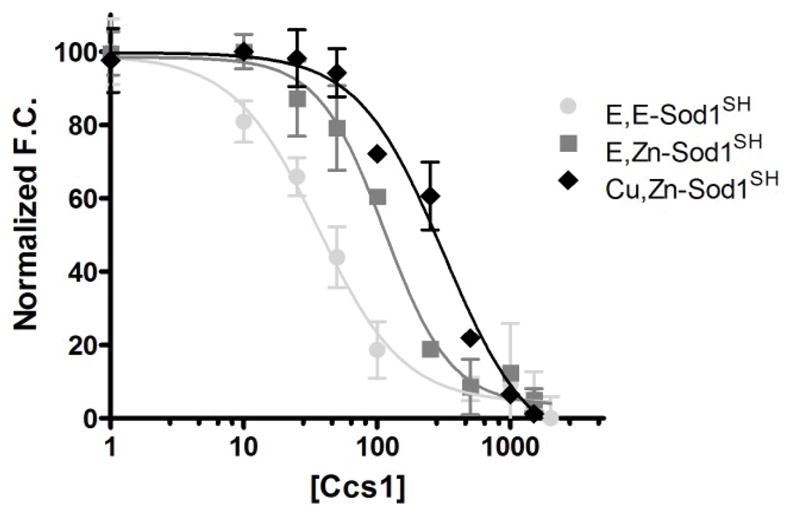
Affinity of Sod1•Ccs1 complex: The H46R/H48Q/C146S Sod1 variant was fluorescently labeled with Alexa-546. The Sod1•Ccs1 interaction is measured using fluorescence quenching. Data was normalized and then fit using a non-linear regression curve for comparison. Binding of Ccs1 to a completely immature (E, E-Sod1^SH^) monomer gives a K_D_ of 34 nM; the E, Zn-Sod1^SH^ dimer binds ~ 4-fold weaker by comparison (K_D_=134 nM). Further post translational modifications (copper binding and disulfide oxidation) to Sod1 continue to decrease the binding affinity even further (K_D_=317 nM).

Quantitative binding assays were performed to determine the affinity of the immature Sod1•Ccs1 interaction. Here, we examined the ability of Ccs1 to bind to Sod1 molecules with increasing post-translational modifications. Ccs1 bound a E, E-Sod1^SH^ form of Sod1 (●) 4-fold tighter than a E, Zn-Sod1^SH^ molecule (■) (K_D_=36 ± 7 nM and 114 ± 16 nM, respectively) (Figure 3), a difference in affinity previously overlooked using less sensitive methods [9]. Addition of copper to the active site of Sod1 (⋄) further diminishes binding and results in an affinity that has decreased 10-fold from the completely immature form (K_D_=371 ± 74 nM). Previous studies have shown that a completely mature from of Sod1 (Cu, Zn-Sod1^SS^), with an oxidized disulfide bond, is no longer recognized by Ccs1 in the concentration range tested (1 nM to 10 μM) (9, 17). Ccs1 clearly prefers a completely unmodified form of Sod1 and raises the question if Ccs1 could play a faciliatory role in proper zinc ion binding by Sod1? We utilize a zinc binding/release assay to test the affinity of Sod1 for zinc when bound and free from Ccs1 (see below) [9,33].

### Ccs1 interaction stabilizes zinc binding by Sod1

**Figure 4:**
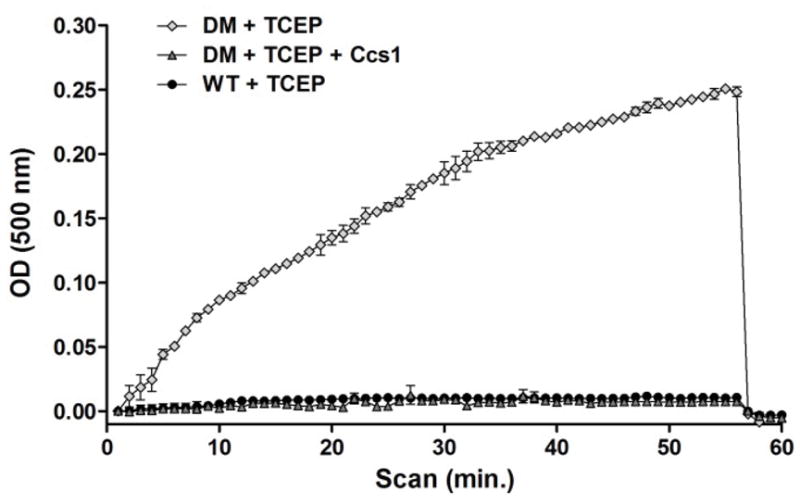
Zinc-release assay: The release of zinc, in the presence of a weak chelator (PAR), from the H46R/H48Q Sod1 mutant (DM) was measured spectroscopically in comparison with wild type (WT) Sod1. The DM cannot bind copper in the active site and leaches zinc upon the addition of PAR, which is exacerbated by the reducing agent TCEP. Ccs1 binding by the disulfide reduced DM Sod1 molecule stabilizes zinc binding and prohibits chelation by PAR.

Copper-free disulfide reduced forms of Sod1 (E, Zn-Sod1^SH^) do not bind zinc with high affinity and the metal can be leached from the protein in the presence of the weak zinc chelator PAR (Figure 4) [9]. Zinc-release from Sod1 can be measured spectroscopically as zinc-bound PAR absorbs light 500 nm. A rapid loss of zinc is seen for a disulfide-reduced form of the active/copper site mutant H46R/H48Q (⋄), which is not seen for the metal replete form of Sod1 (Cu, Zn-Sod1^SH^) (•). This active site variant is used to ensure no metal binding (copper or zinc) within the active site of Sod1, though zinc can still bind at the “zinc-site.” Upon addition of Ccs1 to the disulfide reduced H46R/H48Q mutant (Δ), the ability to retain zinc is restored to that of wild-type Sod1. This suggests that interaction with Ccs1 promotes high affinity zinc binding in Sod1.

### Ccs1 D1 and D3 have roles in high-affinity Sod1 binding

**Figure 5:**
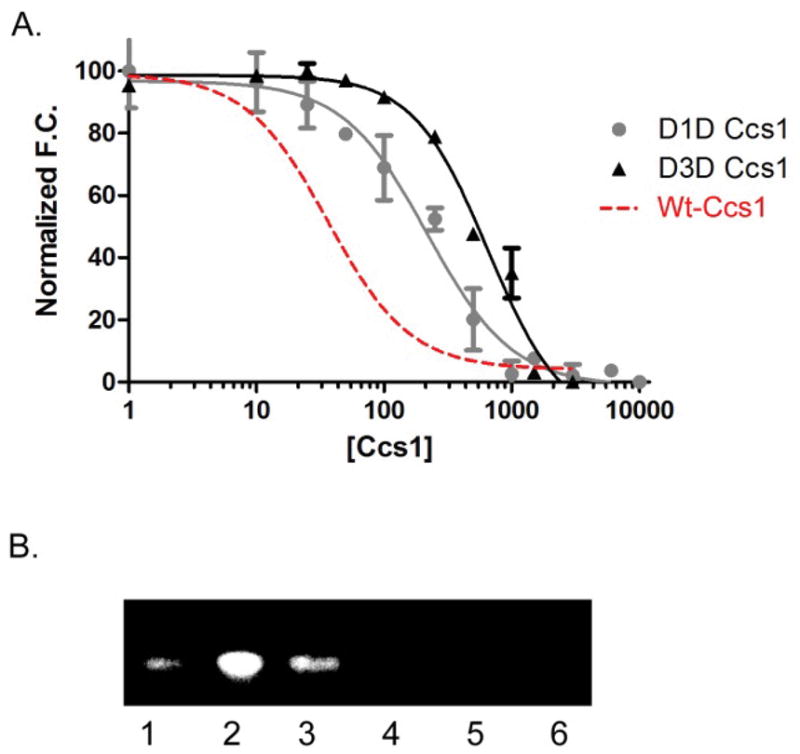
Complex affinity and Sod1 activation with Ccs1 variants: Both truncated forms of Ccs1 lacking either D1 (D1D) or D3 (D3D) show hindered binding with immature Sod1 as compared to the full-length Wt-Ccs1 molecule (red trace) (A). The binding affinity for The D1 truncation (D1D) can bind copper similar to Wt-Ccs1, but shows ~ 5-fold less ability to activate immature Sod1 (B-lane 3). In contrast, the activation capability of the D3 truncation (D3D) has been completely lost (B-lane 4). B-lane 1 is E, E-Sod1^SH^, B-lane 2 is E, E-Sod1^SH^+Cu-loaded yCcs1, B-lane 5 is E, E-Sod1^SH^+apo-yCcs1, and B-lane 6 is H46R/H48Q-Sod1 (cannot bind copper in active site)+Cu-loaded yCcs1.

Ccs1 D2 is structurally similar to Sod1 and targets immature Sod1 monomers for activation. Ccs1 D1 and D3 have confirmed roles during the activation process. We find that both domains also contribute to Sod1 binding either directly or indirectly (Figure 5A). Removal of D1 significantly decreases the Sod1•Ccs1 binding affinity (K_D_=217 ± 38 nM), though structural analysis previously revealed no direct interaction between D1 and bound Sod1. Somewhat similarly, complete removal of Ccs1 D3 has an adverse effect on the binding affinity (K_D_=552 ± 105 nM), though this is likely due to removal of direct interactions between D3 and loop elements within immature Sod1. Truncation of either domain inhibits the ability of Ccs1 to fully activate Sod1 (Figure 5B), though both can bind copper. Data suggests synergistic roles for the Ccs1 domains towards Sod1 binding as a first step towards activation.

## Discussion

**Figure 6:**
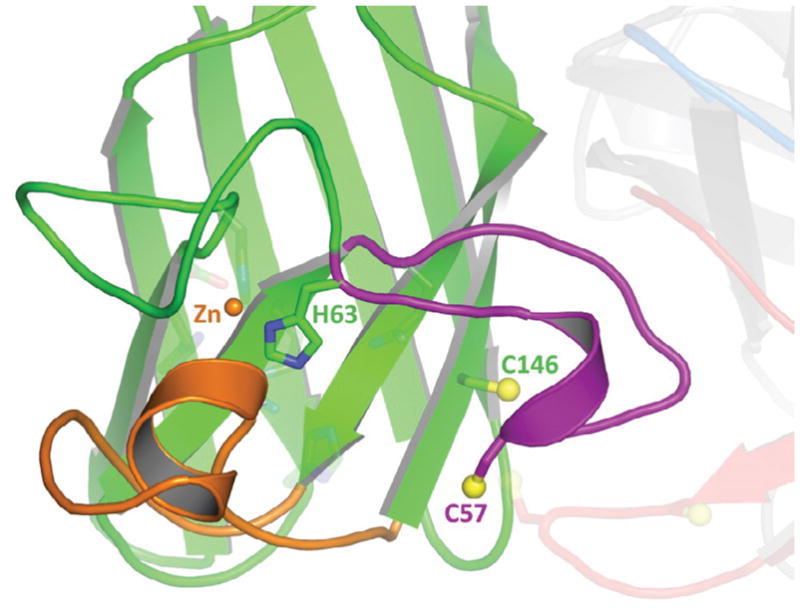
H63 stabilizes the disulfide loop of Sod1 by coordination with zinc in the zinc site: The disulfide loop (purple) and electrostatic loop (orange) of Sod1 are shown with zinc present (orange sphere) in the zinc-site. H63 coordinates the zinc ion and “locks-down” the disulfide loop, which prevents further unfurling by the notch-into-groove interaction from Ccs1 D3 (transparent red). Remaining metal ion liganding residues are shown as transparent sticks.

The data presented here supports new and expanded roles for individual domains within Ccs1. Previous reports suggest singular and individualized roles for the structural Ccs1 domains, though commonly these roles diverged between publications [6,9,12,21,23]. Our data suggests that the domains support function within the other domains, especially regarding recognition and binding of the “substrate” or immature form of Sod1 (E, E-Sod1^SH^) and then release of the “product” or mature form of Sod1 (Cu, Zn-Sod1^SS^). We first employed a structural-based protein engineering technique to site specifically label Sod1 with fluorescent dye [33]. The maleimide linked Alexa-546 dye attached to C57 is productive on two fronts. First, it allows us to monitor the Sod1•Ccs1 interaction at low concentrations (high picomolar at the lower limit) within gels and plates. This is between Sod1 and Ccs1 [31]. Secondly, the attachment at C57 keeps the Sod1 protein in a conformation without a formed disulfide which we have shown previously is essential for recognition with Ccs1 [9]. In all, this provides a new method for the thermodynamic analysis of the Sod1•Ccs1 interaction and activation processes. We then compared the binding of Ccs1 to E, E-Sod1^SH^ and that of E, Zn-Sod1^SH^. The completely immature Sod1 bound Ccs1 4-fold tighter than the zinc-bound disulfide-reduced form (36 nM and 114 nM, respectively). Though each binding event is of relatively high affinity, Ccs1 clearly binds preferentially to the E, E-Sod1^SH^ monomer. This preference could not be elucidated beforehand using less sensitive techniques [9]. In accordance with our recent Sod1•Ccs1 crystal structure, Ccs1 D3 and the disulfide loop of Sod1 create a notch-into-groove interaction that drives the exclusive recognition of disulfide-reduced Sod1 [18]. H63 is a zinc and copper ligand in the Sod1 active site termed the “bridging imidazolate” that resides near the end of the disulfide loop (Figure 6). In the Sod1•Ccs1 crystal structure zinc is bound at the zinc site (H63, H71, H80 and D83), which limits on the dynamic range of the disulfide loop. In a zinc-free disulfide reduced form of Sod1, the disulfide loop would be free to unfurl even further than what is observed in the crystal structure. This conformational freedom may further expand the observed notch-into-groove interaction and strengthen the overall binding affinity. In stark contrast to copper, zinc is relatively abundant in the cell [8]. It is commonly accepted that E, E-Sod1^SH^ acquires zinc diffusively within the cytosol as there are no known zinc metallochaperones. However, we show that an engineered Sod1 active-site variant (H46R/H48Q) cannot bind zinc with high affinity. Bound zinc can be leached out by a weak zinc chelator (PAR) when the Sod1 variant is disulfide reduced [9]. This result is reversed when Ccs1 is bound to the Sod1 molecule indicating that conformational changes that occur to the Sod1 molecule upon binding by Ccs1 promote high affinity zinc binding. This molecular chaperone-like function of Ccs1 directing zinc acquisition by Sod1 is a novel function for Ccs1 or any other metallo-chaperone molecule. In yeast, Ccs1 D1 has a critical role in Sod1 activation, but only under copper limiting conditions [12]. This conditional requirement strongly suggests a role in copper acquisition from the environment. However, structural analysis of our Sod1•Ccs1 complex structure points to a possible role where D1 stabilizes the β-hairpin conformation of D3 that expands the Sod1•Ccs1 interaction interface [18]. This has been suggested qualitatively by others [22] and our binding data backs this up, where deletion of this D1 leads to a 7-fold reduction in the binding affinity (K_D_). This is comparable, but expectedly, not as substantial as truncating D3 from Ccs1, where removal of the entire domain leads to a 15-fold reduction in binding affinity.

## Conclusion

Data presented here supports the notion that the Sod1•Ccs1 interaction is altered during the activation process. Ccs1 recognizes and binds a completely immature form of Sod1 (E, E-Sod1^SH^) (Figure 3) and this interaction induces conformation changes to conserved loop elements in Sod1 that promote high affinity zinc binding (Figures 4 and 6). As we have previously reported, copper is then delivered to an “entry-site” near the Sod1•Ccs1 interface [18]. Delivery by D3 is stabilized via interactions with both D1 and the Sod1 disulfide loop (Figure 5). Sulfenylation at the site leads to disulfide bond formation and copper release to the active site (18). Mature Sod1 (Cu, Zn-Sod1^SS^) can no longer be bound by Ccs1 and prefers interaction with another mature Sod1 monomer [9]. Ccs1 is owed the distinction as a unique multi-use chaperone that promotes three distinct post translational modifications to its target (Sod1).
